# Change in the Order of Activation of Lower Limb Muscles Relative to Spinal Extensors During the Janda Test and Change in Postural Balance in Patients with LBP After Muscle Energy Techniques

**DOI:** 10.3390/jcm14051448

**Published:** 2025-02-21

**Authors:** Katarzyna Wegner-Czerniak, Jacek Mączyński, Anna Błaszczyk, Małgorzata B. Ogurkowska

**Affiliations:** Department of Biomechanics, Poznan University of Physical Education, Królowej Jadwigi 27/39, 61-871 Poznan, Poland; maczynski.j23@gmail.com (J.M.); annasz1@onet.pl (A.B.); ogurkmal@man.poznan.pl (M.B.O.)

**Keywords:** muscle energy techniques, electromyography, posturography, Janda test

## Abstract

**Background:** The aim of this study was to investigate change in the order activation of leg muscles in relation to the spinal extensors during the Janda test and change postural balance in patients with low back pain (LBP) after muscle energy techniques (METs). **Methods:** The study included fifteen men (mean age 41.9 years) working on an assembly line. The activity and recruitment of the following muscles: biceps femoris (BF), gluteus maximus (GM) and erector spinae (ES) were measured with the use of surface electromyography (sEMG) during the prone hip extension (PHE) test. Pain levels and postural balance were analysed. **Results**: MET translated into a reduction in muscle activation time in both the left and right side of the body (*p* < 0.001). The change was observed in the following muscles: BF (*p* = 0.003) and GM (*p* = 0.004). A reduction in pain was obtained post application of the MET therapy (*p* < 0.001). It was observed that after the therapy, along with the later activation time of the GM muscle, the range of motion of the COP along the *x*-axis increased (*p* = 0.0368). Increased activation time of the RES (*p* = 0.0411) and the LES (*p* = 0.0350) muscles influenced a decrease in the COP range of motion along the *x*-axis. **Conclusions**: The use of MET in people with LBP improves the sequence of activation of the ES, GM and BF muscles, affects postural balance, allows for the restoration of muscle balance in the lumbar spine and lower limbs, and reduces pain.

## 1. Introduction

Low back pain (LBP) represents a common and often debilitating condition that encompasses a range of disorders, characterised by discomfort in the area between the 12th rib and the iliac crest. Notably, in 2020, approximately 619 million individuals worldwide were affected by LBP, accounting for nearly 10% of the global population. This figure is projected to rise to 843 million by 2050, highlighting its status as a global health challenge often referred to as the epidemic of low back pain [1]. In industrialised countries, about one-third of all health-related absences from work are caused by musculoskeletal disorders. Most of these disorders, i.e., about 60%, are caused by LBP [2].

A review of working conditions in Europe leads to the conclusion that forced body posture (non-ergonomic, task-specific) and repetitive movements rank first and most important on the list of risk factors for overload syndromes in the work environment. As many as 45% and 60% of employees are exposed to these factors for more than 25% of their daily work time [3]. The nature of the work of an assembly worker is mainly based on performing repetitive movements in forced body positions. In one day, 270 cars are produced. The specificity of work on the assembly line forces repetitive forward bending and straightening movements when reaching for structural elements. Long-term loads resulting from several years of unilateral movement are the basis for the statement about the occurrence of functional overload changes in the musculoskeletal system among people working as assembly workers [4]. In the production process, the human factor has a major impact on productivity, costs and the quality of production. In European countries, the total costs associated with LBP range from 0.1 to 2% of the gross domestic product [5,6].

In order to prevent musculoskeletal dysfunctions, unnecessary mechanical overload should be avoided and mobility and balance should be increased in order to achieve a greater load tolerance [7]. Exceeding the physical endurance or functional capacity of muscles, bones and joints leads to overload changes. Since the stabilising system (muscles) becomes inefficient due to overloading, loads are transferred to the passive stabilising system, i.e., the ligaments and capsules of the intervertebral joints. If the overloading movement continues to be repeated, an increase in the shear component of the resulting force acting on the intervertebral disc causes damage to the intervertebral disc and subsequent morphological changes in the vertebral bodies and intervertebral joints [8].

Low back pain (LBP) is a complex health issue with significant clinical relevance. Advances in understanding the pathophysiology of intervertebral discs have provided new insights into the multifactorial causes of discogenic pain. Despite these advancements, diagnostic challenges persist due to symptom overlap with other conditions and the lack of definitive diagnostic criteria. According to Simone et al., artificial intelligence (AI) holds the potential to significantly impact the diagnosis and treatment of LBP. Its application in diagnostic imaging, medical record analysis, and surgical planning has shown promising results. Integrating clinical data with AI-based technologies could play a key role in improving LBP management strategies [9]. One of the treatment options is manual therapy, which, in cases of chronic LBP, focuses on reducing pain by restoring the range of motion in the spinal joints or adjacent structures, assuming that joint restriction may contribute to the pain [10]. The literature features a number of comparative analyses of the various therapeutic approaches applied in the treatment of LBP. Evaluation of the effectiveness of a therapy is often based on the subjective feelings of patients [11].

Muscle energy techniques constitute a group of osteopathic (originally) soft tissue manipulation methods. Their aim is to reduce pain by using isometric tension and/or isotonic movements performed by the patient. The movement is precise and performed in a strictly defined position, adjusted to the degree and type of dysfunction [12]. The result is improved spinal mobility after a single session as well as after a four-week therapeutic cycle [13,14]. The results of these studies are based only on the use of a measuring tool, which is a goniometer.

It has been shown that people with LBP have reduced awareness of the direction of movement and position of the lumbar spine [15]. It is suspected that intervertebral lesions that activate nociceptors located in any structure within the spine will stimulate nociceptive pathways. Moreover, they may lead to a reduction in the amount of afferent proprioceptive information from such a segment. On top of that, LBP may cause changes in the activity of the paraspinal muscles, inducing an overreaction to the stimulus on the part of superficial muscles, while inhibiting the action of the paraspinal muscles located deeper. Consequently, local proprioceptive disorders and changes in motor control may affect segmental balance and cause further overload. Chaitow et al. [12] suggest that the benefits of manual therapy treatments may go beyond short-term effects on proprioception and motor control. However, there is no research in this area using MET.

Based on his clinical observations, Janda [16] suggested that the prone hip extension (PHE) test could be used to assess functional muscle imbalance. Furthermore, he believed that it was important in the development and/or perpetuation of LBP. However, the PHE test is not often used clinically because of difficulties in the diagnosis made by the physician or therapist regarding the sequence of muscle activation. It requires a lot of palpation experience on the part of the clinician. Moreover, capturing small changes in muscle activity with a subjective measurement method seems highly questionable. Therefore, the purpose of this study was to investigate using surface electromyography whether there is a change in the order of GM and/or BF activation relative to ES in the Janda test after MET therapy in patients with LBP. In addition, the relationship between changes in muscle activation and patients’ postural balance was assessed.

## 2. Material and Methods

The study included fifteen men (mean age 41.9 years ±6.8 SD, mean BMI 27.8 kg/m^2^ ±2.1 SD) with chronic non-specific low back pain syndrome working on an assembly line in the automotive industry (Table 1). The work of an assembler to a large extent involves repetitive movements and adopting non-ergonomic body positions [4].

The tests were approved by the Bioethics Committee at Poznan University of Medical Sciences (approval no 623/15). All the subjects expressed consent in writing to participate in the project. All procedures were compliant with the Declaration of Helsinki of 1964.

### 2.1. Experimental Procedures

#### Participants

Inclusion in the study group was based on a biomechanical analysis of the workstations. The initial classification of patients was based on a questionnaire. The inclusion criterion was lumbar pain of at least 7 points or higher on the Visual Analog Scale (VAS) lasting more than 12 weeks [17]. It was important that the subjects reported severe and chronic pain that affected the functional state of the musculoskeletal system and worsened during professional work. The orthopedist selected fifteen patients based on the questionnaire and physical examination and referred them for a computed tomography (CT) scan of the lumbar spine. Intervertebral disc protrusion at the L1-L2 level was diagnosed in 10% of the men, L2-L3—in 15%, L3-L4—in 17%, L4-L5—in 28%, and L5-S1—in 30%. The following exclusion criteria were established: lumbar spine surgery, diagnosed spondylolisthesis, neoplastic disease, spinal fractures, female gender.

### 2.2. Biomechanics Measurements

The selected men were required to participate in two independent examination sessions during which the recruitment and activation time of the muscles of the lumbopelvic region (left and right erector spinae muscles, right and left gluteus maximus muscles and right and left biceps femoris muscles) were assessed during the PHE test with the use of sEMG.

### 2.3. sEMG Measurement

The sEMG method enables the assessment of the relationship between the temporal parameters of muscle activity and the performed movement [18]. The aim of the PHE test was to assess the effectiveness of the proposed therapy. The order of activation during the test was determined with reference to the BF muscle, which according to Janda’s standards should be activated first. The second muscle for the left lower limb should be the GM, then RES (right erector spinae), and the last LES (left erector spinae). The activation of the ipsilateral ES in the context of the lifted limb should be the last. In the right lower limb, muscle recruitment in the PHE test was as follows: BF, GM, LES, RES [19].

**Figure 1:**
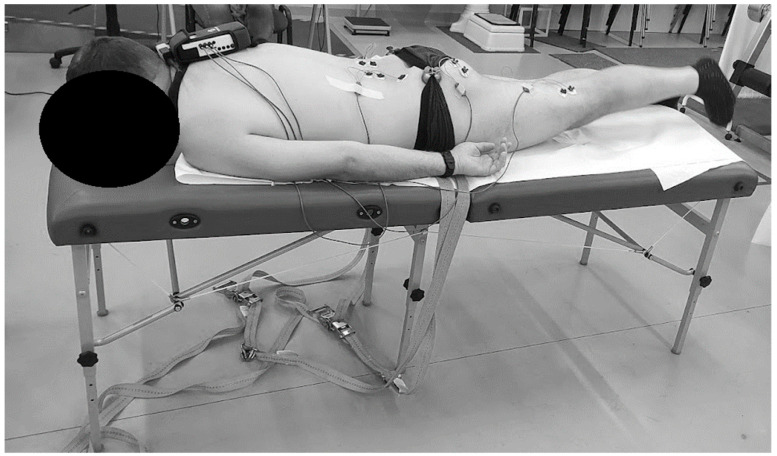
sEMG measurement during the PHE test.

Prior to the test, the patients did a warm-up on a cycloergometer each time. As instructed by the person conducting the examination, the patient performed hip extension (ca. 20–30 degrees) of the lower limb, first the right one, then the left one, in the prone position (Figure 1). The patient maintained this position for three seconds and then returned to the initial position. Five PHE tests were performed for each limb with simultaneous analysis of the electromyographic signal. The procedure was performed according to the SENIAM recommendations. Self-adhesive electrodes were positioned as follows: for ES—bilaterally 2 cm from the spinous processes of the L3 vertebra region; for GM—in the middle of the line connecting S2 with the greater trochanter; for BF—laterally in the middle of the line between the buttock fold and the popliteal fossa. The sequence and timing of muscle activation were measured. The sEMG measurement was performed using a 16-channel TeleMyo 2400T G2 telemetric sEMG system. According to the SENIAM recommendations, fifteen disposable, self-adhesive Ag/AgCl electrodes (SORIMEX, Poland, 1 cm diameter) were placed parallel to the course of the muscle fibres. Signal processing was performed with the use of the MyoResearch XP Master Edition software (Noraxon, AZ, USA) [20].

### 2.4. Posturographic Tests

Postural balance was tested before and after the therapy. A single-module AccuSway stabilometric platform from AMTI with the Balance Clinic software package was used. The subjects underwent measurements for balance in four variants: standing on both feet with the upper limbs along the body, eyes open and closed, and standing on one leg—left and right—with eyes open. The men were unable to perform the single-leg standing test with eyes closed. The subjects’ task was to remain as still as possible for 30 s in the two feet test and for 15 s in the single-leg test. The subjects performed 3 tests for each of the four balance measurement variants. Intervals between the measurements lasted 60 s. The analysed parameters obtained in the balance test were the mean COP (centre of pressure) velocity, the ranges of motion of the COP in the frontal and sagittal planes, and the area of the ellipse comprising 95% of the measurement points. One of the three tests with the lowest COP velocity, i.e., with the best balance, was selected for statistical analysis.

### 2.5. Therapeutic Methods

The therapy consisted of three osteopathic MET sessions with a seven-day break in between. The therapy was performed by a certified osteopath who obtained the D.O. title after completing 1500 h of postgraduate osteopathic training and also having a master’s degree in physiotherapy. In the segmental MET methodology, the most important thing is to recognise the limiting barrier in the case of joint dysfunction. In the study group, a resistive contraction lasting from 3 to 7 s was used according to the Mitchell guidelines [21]. Patients were asked to keep the contraction at 20% of the maximum force they could generate. The osteopath palpated the tension of the paraspinal muscles at a selected level of the spine and thus assessed the strength of the contraction. He corrected the force used by the patient through verbal commands. The first phase of the therapy involved palpation of the spinous processes of the lumbar spine and the spinous process of S1. In the next phase, the therapist performed passive rotation movements and side bends to determine the mobility deficit. The spine mobility barrier on both sides was compared to correctly identify the disorder. The patient was lying in the supine position. Depending on the type of dysfunction diagnosed, two versions of mobility on a given spinal segment were used. The first version denotes the diagnosis of ERS-right, i.e., lumbar vertebra mobility deficit during forward bending, left rotation, and left lateral bending. The same technique can be used to normalise the ERS-left dysfunction, in which case the patient should be placed on the right side and the parameters should be reversed. The second version for FRS-left indicated lumbar vertebra mobility deficits in extension, left rotation, and left lateral bending. The same technique enables normalisation of the FRS-right dysfunction, in which case the patient should be placed on the left side and the parameters should be reversed [22]. Each therapy began with repeated diagnosis of the level and type of dysfunction, so that the techniques were selected in line with the patient’s current functional status. The duration of each session was approximately 30 min. After the therapy, patients underwent another examination in a biomechanical laboratory.

Before and after the three-week treatment cycle, the men completed a short questionnaire. They determined the pain scale and marked the location of the pain on the spine on a drawing, taking into account possible radiation to the lower limb.

## 3. Statistics

Statistical calculations were performed using the TIBCO Software Inc. (2017) Statistica (data analysis software system), version 13 (Santa Clara, CA, USA). Normality of data distribution was verified using the Shapiro–Wilk test. Differences in muscle activation time before and after MET were calculated using multivariate ANOVA. Tukey’s honest significant difference (HSD) test was used to identify specific differences. In the case of statistically significant differences between muscle activation times and terms, multidimensional 4 × 2 ANOVA (muscle × term) was used, and for imbalances in muscle activation times, 4 × 2 ANOVA (limb × term) was performed. The level of statistical significance was set at *p* < 0.05. Spearman correlations of times of the muscle activation onset with posturographic parameters were also established.

## 4. Results

### 4.1. Analysis of sEMG Parameters

**Figure 2:**
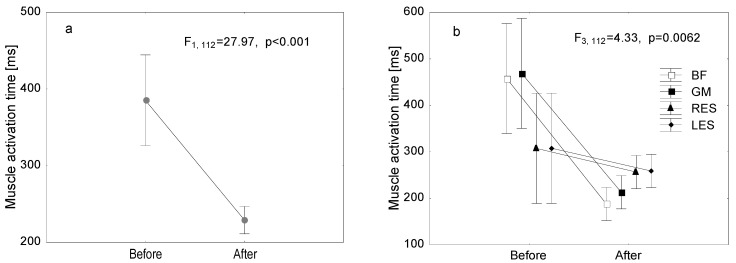

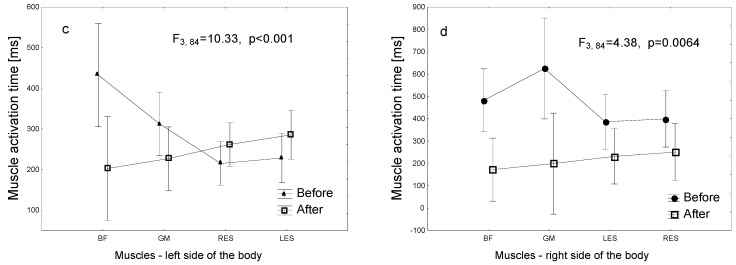
Muscle activation time before and after MET ((**a**) general; (**b**–**d**) for each muscle separately). Mean values with 95% confidence intervals are shown.

The aim of the EMG examination was to test whether METs improve the sequence of muscle activation in LBP patients during the PHE test. A highly statistically significant (*p* < 0.001) decrease in the muscle activation time of both the left and right side of the body was found for all the muscles (BF, GM, RES, LES) after the therapy (Figure 2a). Furthermore, an interaction was found between the activation time of individual muscles and the date of the test (pre-therapy and post-therapy) (4 × 2) (*p* = 0.0062) (Figure 2b). A highly significant reduction in activation time for both BF muscles was found (*p* < 0.001). In the case of the GM muscles (left, right), it was noted that the electrical activation onset time was highly statistically shorter after the treatment (*p* = 0.0038). The faster activation of the GM muscle in the right limb was statistically highly significant (*p* = 0.0045). Moreover, for the right limb, the GM activation time was statistically significantly longer than the activation time of the LES (*p* = 0.0078) and RES (*p* = 0.0148) muscles (before therapy) and the BF (*p* = 0.0038), LES (*p* = 0.0178) and RES (*p* = 0.0285) muscles (after therapy).

An interaction between the activation time of individual muscles and the timing of the test (pre-therapy and post-therapy) (4 × 2), separately for the left (*p* < 0.001) (Figure 2c) and right (*p* = 0.0065) lower limb (Figure 2d), was also found.

**Figure 3:**
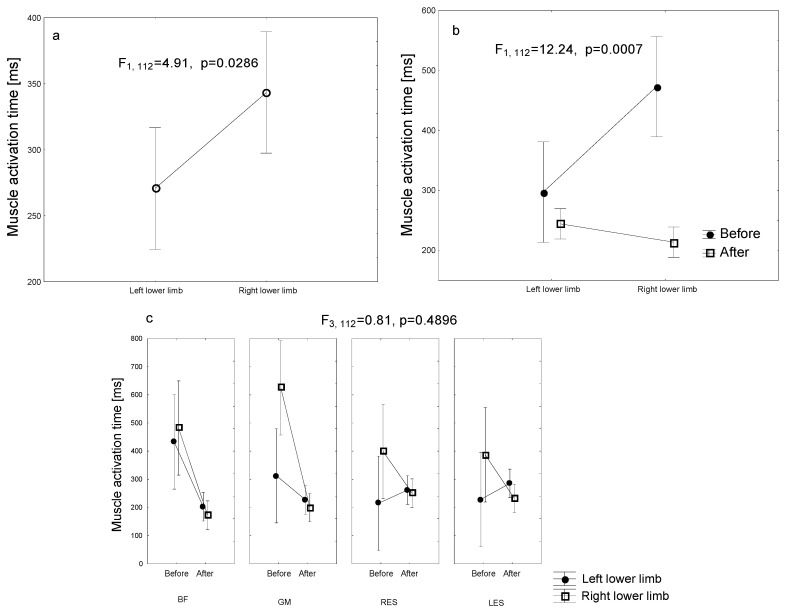
Muscle activation time for the left and right lower limbs ((**a**,**b**) general; (**c**) for each muscle separately). Mean values with 95% confidence intervals are shown.

The study also tested whether there was a difference in the time of onset of muscle EMG activity (BF, GM, RES, LES) between the left and right side of the body. An imbalance in the time of onset of activation was found for all the muscles tested when performing the PHE test, that is, a statistically significantly longer electrical activation time (*p* = 0.0286) occurred on the right side of the body (Figure 3a). Furthermore, there was an interaction between the imbalance in muscle activation time and the time of measurement (before and after therapy) (2 × 2) (*p* < 0.001) (Figure 3b). Before the therapy, the left side of the body had a statistically significantly shorter activation onset time of the tested muscle group, compared to the right side (*p* < 0.001). The largest significant difference was observed in the GM muscle (*p* = 0.0097). After the therapy, there was also a highly statistically significant reduction in the GM reaction time on the left side of the body (*p* < 0.001). Furthermore, the post-therapy activation time of the left GM muscle was significantly lower than that of the right GM before therapy (*p* = 0.0012). During the right leg lift in the PHE test, there was a statistically significantly longer RES muscle activation time before the therapy, compared to the right leg lift (*p* = 0.0397). Moreover, after the therapy, there was a tendency towards a significantly lower RES activation time during the left leg lift, compared to the right leg (*p* = 0.0623). There was a trend towards a statistically significantly longer LES muscle activation time before the therapy, compared to lifting the left leg during the PHE test (*p* = 0.0993). After treatment, there was a trend towards a significantly lower LES activation time during the left limb lift (*p* = 0.0519) (Figure 3c).

### 4.2. Analysis of Balance Parameters

Table 2 shows the correlations between BF, GM, LES and RES muscle activation times and the XR, YR and AE posturographic parameters. It was found that date before the therapy, the above relationships were statistically significant, particularly in the left lower limb. The AE increased significantly (*p* = 0.0462) together with an increase in the BF muscle activation time (left limb) while standing on both feet with eyes closed. In the left leg standing test, the COP range of motion in the sagittal plane decreased together with an increase in the BF (0.0444) and LES (0.0134) reaction time. Furthermore, the AE value when standing on the left leg decreased together with an increase in the activation time of the LES muscle.

After the therapy, during the two feet standing test with eyes open, the COP range of motion in the frontal plane increased statistically significantly together with an increase in the activation time of the GM muscle (*p* = 0.0368). Furthermore, when standing on the right leg, the LES (*p* = 0.0411) and RES (*p* = 0.0350) muscle activation times were found to have a statistically significant effect on decreasing the COP range of motion in the frontal plane.

Following the therapy, the level of pain measured on the VAS decreased highly statistically significantly from 7.5 to 2.3 (*p* < 0.001). Furthermore, in the pre-therapy questionnaire, the patients were asked to indicate the location of their pain along with information on radiation to the limbs. Ten out of the fifteen men experienced pain in the left limb and five only experienced pain centrally. None of the respondents complained of pain in the right lower limb.

## 5. Discussion

The most important result of this study is the confirmation of the beneficial effect of osteopathic METs on changing the order of activation of the GM and BF in relation to ES in the Janda test after MET therapy in patients with LBP. The relationship between changes in muscle activation and postural balance in patients was also confirmed. There are many studies in the literature stating the efficacy of MET [5,23,24,25,26]; however, most of them are based on subjective assessment of pain according to the VAS or ODI index. Furthermore, to our knowledge, there are no scientific publications evaluating the effectiveness of segmental muscle energy techniques in patients with LBP based on sEMG images. In our study, the applied therapy was proven to have a statistically significant effect on improving the activity of lower limb muscles and erector spinae muscles. In the studied group of LBP patients, before the therapy, there was a delayed activation of the gluteus maximus and biceps femoris muscles, compared to the erector spinae muscles. This confirms Levit and Chaitow’s theory that, in individuals with normal motor control, lower limb muscles should activate before erector spinae muscles during hip extension movements [10,12]. The therapy applied in the studied group resulted in faster activation of gluteus maximus and biceps femoris muscles during the PHE test. It should be noted that there is no consensus in the literature as to the order of muscle recruitment established by Janda. A study on an asymptomatic group and a group with LBP showed that there was a delay in gluteal muscle activation during the PHE test in individuals with pain [27,28]. In our study, there was a delayed activation of the gluteal muscles during hip extension movement with simultaneous occurrence of LBP. Coopera also states that there is a decrease in gluteal muscle activity in LBP patients [29]. MET resulted in an increase in the activity of these muscles, relieving stress on the paraspinal muscles. It has been shown that equalising the tension of paraspinal muscles increases the activation of lower limb muscles. According to Bourdillon [30], muscle shortening may be an automatic mechanism resulting from an overreaction of the central system gamma. It is suspected that as long as this mechanism is operating, the muscle cannot return to its normal resting length. Pain occurs due to the inability of the muscle to return to the desired anatomical length. Thanks to the proposed research method, we know that affecting the distal muscles of the lower limb is possible by acting on the spinal joints through the short paraspinal muscles. Eliminating tension at the level of the lumbar spine affects the tension of the posterior myofascial chain. Spinal pain is relieved thanks to faster muscle activation. In addition, active spine stabilizers are supported by the distal muscles of the lower limbs.

The long work experience (over twenty years) of the studied men probably contributed to the pathological changes in the L1-S1 spinal segment diagnosed by the CT scan. The specificity of the work in this plant entails repetitive movements in the sagittal plane when reaching for construction elements. The flexion and extension movement of the lumbar spine with the load causes pressure change in the intervertebral disc and displacement of the nucleus pulposus to the back [31]. The radiation of pain to the left limb may be caused by the asymmetry of loading in the frontal and transverse planes in the course of performing assembly line work. Before the treatment, a relationship between the order of muscle activation in the left limb and postural balance was observed. An increase in the reaction time of the erector muscles and a faster activation of the muscles of the left lower limb (BF) had a positive effect on the subjects’ balance. After the therapy on the right limb, longer response time on the part of the erector spinae muscles resulted in improved postural balance parameters. Some researchers emphasise that the ability to maintain balance depends on muscle strength and the speed at which the muscle shortens [32,33].

The right limb without pain symptoms responds better to therapy. When comparing pre-treatment reaction times for both gluteal muscles, a significant delay was observed on the right side. This is suspected to be related to the overloading of the right gluteal muscle. There was an instinctive reduction in strain on the lower limb which was hurting. In the case of the left limb, the stabilising function of the pelvis was taken over by the gluteus maximus muscle on the opposite side together with the other gluteal muscles and fascia. The most important function of the gluteus maximus muscle is to maintain a vertical posture while standing and walking. It is one of the stabilising (postural) muscles responsible for the correct posture of the entire body [34]. This is also confirmed by the above study: the reduction in GM work time achieved by MET translates into a reduction in the range of movement of the COP in the frontal plane. After the therapy, there was an equalisation of the tension of both gluteal muscles in relation to each other. Lumbar spine muscle manipulation improved pelvic stabilisation. This is particularly important for this occupational group, which performs most of its working time while standing with pelvic anteversion.

Jabłońska et al. [35] proposed evaluating the McKenzie therapy on the basis of sEMG as well. Muscle recruitment and pain intensity decreased following exercise according to the McKenzie method. A faster reaction time of the BF and GM muscles during the PHE test was observed. The therapy lasted only seven days, the patients performed it independently and unsupervised, and the results are presented for the right lower limb only. Muscle recruitment results are presented as a percentage. No data on the statistical significance of the results are available. In our study, with the use of MET, the improvement in BF and GM muscle activation was statistically significant when compared to the percentage of improvement in the McKenzie therapy. However, it should be noted that both the McKenzie therapy and MET have a statistically significant effect on pain reduction in patients with LBP. Jabłońska’s paper [35] lacks data on the radiation of spinal pain to the lower limb. This is important information because, based on the results of our study in the lower limb without pain symptoms, the timing of muscle activation and muscle recruitment is significantly different from the limb with symptoms. The McKenzie therapy focuses on pathology within the intervertebral discs by mechanically applying pressure on the nerve root, thereby resulting in a significant reduction in pain [36]. MET, on the other hand, focuses on the myofascial system as a source of movement limitations and compensatory mechanisms of the musculoskeletal system. Better results of MET are due to the fact that, according to Schlenk et al. [37], neglecting muscles surrounding joints, especially spinal joints, in the treatment of joint dysfunctions is a mistake. Paying attention to the supporting function of muscles will normalise these muscles without excessive effort. Therefore, methods focusing on soft tissues, such as MET, allow for the correction of both weaker and shortened muscles, often fibrous antagonistic muscles. The above studies confirm the relationships observed by Cana-Pino et al. that with the reduction in CoP displacement the pain decreases in patients with LBP after the intervention as well as after a 3-month follow-up [38].

Szulc et al. [39] in their study proved that the use of MET in combination with the McKenzie therapy has a more beneficial effect on spinal mobility than the McKenzie therapy alone, thereby confirming that it is myofascial limitations that affect spinal mobility. They demonstrated that the use of MET is safe and does not adversely affect the amount of disc herniation on MRI. This confirms the validity of using these techniques in people with intervertebral disc pathology.

### 5.1. Clinical Implications

These results may represent a change in osteopathic treatment planning since deficits in postural control are not usually considered in the treatment of LBP. Diagnosis of the level and type of dysfunction used in MET enables restoration of muscle balance in the lumbar spine and lower limbs. As these studies have shown, working on the spine area improves distant structures, i.e., the gluteal muscles. Evaluation with technological devices makes the results objective, which provides therapists with greater objectivity in the subjective variables that patients indicate to us, such as the VAS or ODI scale.

### 5.2. Limitations of the Study

The main limitation of this study is the lack of a control group that did not receive the intervention. This would allow for a comparison of effects and whether time had no effect on the healing process. However, the use of simulated therapy in adults is difficult, unlike in the case of pharmacology where a tablet with placebo can be administered. Putting the patient in a simulated position and asking them to tense their muscles, for example, in an uncontrolled manner, could worsen their condition. In the case of chronically ill people with long-term work experience, a spontaneous healing process seems unlikely. Future research is needed to examine the long-term effects of the therapy and analyse the observed changes, how long they last over time and whether strengthening the gluteal muscles together with MET would bring long-term improvement in the patient’s condition.

## 6. Conclusions

The use of segmental muscle energy techniques in individuals with LBP improves the activation sequence of the ES, GM and BF muscles, which has an impact on postural balance and reduction in pain. The prevalence of LBP should encourage the selection of the most effective treatment methods, taking into account not only the reduction in pain but also a change in the myofascial system influencing the delay of overload changes within the intervertebral discs.

## Figures and Tables

**Table 1:** Anthropometric parameters (N = 15).

Parameters	Mean ± SD	Minimum	Maximum
Age [years]	41.9 ± 6.8	31.0	55.0
Body height [m]	1.80 ± 0.06	1.70	1.93
Body mass [kg]	90.1 ± 7.6	80.2	106.7
BMI [kg/m^2^]	27.8 ± 2.1	24.4	31.6
Work experience [years]	22.2 ± 3.8	10.2	29.7

**Table 2:** Spearman correlations of muscle onset activation times with posturographic parameters (PPs). N = 15.

	PP&Position	T	Body Side	R	*p*-Value
First examination					
1	AE EcCb	BF	L	0.5214	0.0462
2	YR EoSl	BF	L	−0.5250	0.0444
4	YR EoSl	LES	L	−0.6214	0.0134
5	AE EoSl	LES	L	−0.6178	0.0141
Second examination					
8	XR EoCb	GM	R	0.5420	0.0368
9	XR EoSr	LES	R	−0.5321	0.0411
10	XR EoSr	RES	R	−0.5464	0.0350

XR (YR): range of movement of the COP in the frontal (sagittal) plane. AE: area of the ellipse containing 95% of the COP points. EoCb: Eyes open Closed base. EcCb: Eyes closed Closed base. EoSr: Eyes open Single right. EoSl: Eyes open Single left. The coding of muscles is as follows: (BF, GM, ES) (L or R—left or right). ES (L or R—left or right leg raised).

## Data Availability

The datasets used and/or analysed during this study are available from the corresponding author upon reasonable request.
